# *Microbacterium altimontanum* sp. nov., *Microbacterium longlingense* sp. nov., and *Microbacterium cucumeris* sp. nov.: Three New Species of *Microbacterium* Isolated from Soil in Yunnan Province, China

**DOI:** 10.3390/microorganisms14081644

**Published:** 2026-07-28

**Authors:** Wenqi Lai, Xiankun Zhang, Zhongwen Duan, Mingji Yang, Yongxia Wang, Zefen Yu

**Affiliations:** 1 State Key Laboratory for Conservation and Utilization of Bio-Resources in Yunnan, Yunnan University, Kunming 650504, China; laiwenqi2024@163.com (W.L.); 15368117743@163.com (X.Z.); 18348057785@163.com (Z.D.); 15008716509@163.com (M.Y.); 2 Yunnan Key Laboratory of Basic Research and Innovative Application for Green Biological Production, Yunnan University, Kunming 650504, China; 3 School of Life Sciences, Yunnan University, Kunming 650504, China; 4 Yunnan Institute of Microbiology, School of Life Sciences, Yunnan University, Kunming 650504, China

**Keywords:** *Microbacterium*, soil, polyphasic taxonomy, genome analysis

## Abstract

Three actinobacterial strains, AW10-10^T^, M8-1^T^ and HGHZ1-2^T^, were isolated from soil in Yunnan Province, China. Analyses of 16S rRNA gene sequences and shotgun-sequenced draft genomes placed them within the genus *Microbacterium*. OrthoANI, FastANI and dDDH values between the three strains and their closest relatives were 75.7–79.6%, 79.9–81.9% and 19.7–22.5%, respectively, all well below the accepted species-level thresholds. Cells of all three strains were Gram-stain-positive, aerobic and rod-shaped. The major fatty acids were anteiso-C_17:0_, anteiso-C_15:0_ and iso-C_16:0_ in AW10-10^T^ and HGHZ1-2^T^, and anteiso-C_15:0_, anteiso-C_17:0_ and iso-C_16:0_ in M8-1^T^. AW10-10^T^ and M8-1^T^ contained ribose, galactose, mannose, glucose and minor rhamnose, whereas HGHZ1-2^T^ lacked rhamnose. The predominant menaquinones were MK-12 and MK-13 in AW10-10^T^, MK-13 and MK-12 in HGHZ1-2^T^, and MK-10, MK-11 and MK-9 in M8-1^T^. Polar lipids comprised diphosphatidylglycerol, phosphatidylglycerol and one unidentified glycolipid in AW10-10^T^ and HGHZ1-2^T^, but two unidentified glycolipids in M8-1^T^. DNA G + C contents were 70.0, 69.7 and 69.5 mol%, respectively. The strains represent three novel species, *Microbacterium altimontanum* sp. nov., *Microbacterium longlingense* sp. nov. and *Microbacterium cucumeris* sp. nov., with type strains AW10-10^T^ (=GDMCC 16749^T^ = KCTC 59693^T^), M8-1^T^ (=GDMCC 16752^T^ = KCTC 59696^T^) and HGHZ1-2^T^ (=GDMCC 16751^T^ = KCTC 59694^T^), respectively.

## 1. Introduction

The genus *Microbacterium* was originally proposed by Orla-Jensen in 1919, with *Microbacterium lacticum* as the type species, and the description of the genus was subsequently emended to accommodate an increasingly diverse range of phenotypic and chemotaxonomic characteristics [1,2,3]. Members of the genus are generally aerobic, Gram-stain-positive and non-spore-forming bacteria, with cells ranging from coccoid forms to short or irregular rods; depending on the species, cells may be motile or non-motile [4]. Species of *Microbacterium* are characterized by unsaturated menaquinones containing 9–14 isoprene units and by predominantly iso- and anteiso-branched cellular fatty acids, particularly anteiso-C_15:0_, anteiso-C_17:0_ and iso-C_16:0_ [3,5,6]. Although the genus is generally well supported by 16S rRNA gene-based phylogenetic analyses, genome-scale studies have revealed considerable intrageneric diversity and complex phylogenomic relationships [6].

Members of the genus *Microbacterium* have been isolated from diverse habitats, including soils, plant-associated environments, marine ecosystems and anthropogenic environments such as waste landfills [6,7,8,9,10]. Among these habitats, soils and plant-associated environments have repeatedly yielded novel species, indicating that the taxonomic and genomic diversity of soil-associated members of the genus remains incompletely characterized. The isolation and genome-based characterization of additional soil-derived lineages can therefore improve species-level resolution within this phylogenomically diverse genus and provide reference material for comparative genomic studies.

Beyond their broad ecological distribution, strains of *Microbacterium* exhibit a range of potentially valuable biological functions. Some plant-associated strains have been reported to produce indole-3-acetic acid, solubilize phosphate or exhibit 1-aminocyclopropane-1-carboxylate deaminase activity [7,11,12]. Root-associated strains can also release volatile compounds that modify plant growth and root development [11]. In particular, volatiles produced by *Microbacterium* strains increased the biomass of *Arabidopsis thaliana*, lettuce and tomato and influenced plant sulfur and nitrogen metabolism. Other members of the genus have been reported to produce bioemulsifiers and industrially relevant enzymes and to participate in heavy-metal removal and biotransformation, indicating their potential applications in biotechnology and environmental remediation [13,14,15]. These ecological and functional characteristics make the discovery and taxonomic characterization of novel *Microbacterium* species important for understanding bacterial diversity and identifying microbial resources with potential biotechnological applications.

The 16S rRNA gene remains a useful marker for the preliminary phylogenetic placement of prokaryotic strains. However, 16S rRNA gene sequence identity ranges overlap among taxonomic ranks, and sequence identity alone may not provide reliable species delimitation in all cases [16,17]. Contemporary prokaryotic taxonomy therefore increasingly relies on whole-genome sequence data and overall genome-relatedness indices (OGRIs), particularly average nucleotide identity (ANI) and digital DNA–DNA hybridization (dDDH) [18]. ANI values below approximately 95–96% and dDDH values below 70% generally support the separation of two strains at the species level [18,19,20]. Nevertheless, these genomic indices should be evaluated together with phylogenomic relationships and phenotypic and chemotaxonomic characteristics when determining the taxonomic status of putative novel species.

Strains AW10-10^T^, M8-1^T^ and HGHZ1-2^T^ were isolated from soil samples collected in Yunnan Province, China, during a cultivation-based investigation of culturable bacteria. Their taxonomic positions were investigated using a polyphasic approach integrating phenotypic and chemotaxonomic characterization, 16S rRNA gene phylogeny, phylogenomic analysis, ANI and dDDH comparisons. Comparative genome analyses were additionally conducted to assess genome-wide divergence and to survey predicted biosynthetic gene clusters, prophage regions and other strain-specific genomic features. The combined evidence supports the recognition of the three strains as novel species of the genus *Microbacterium* and provides reference strains and genome sequences for future comparative studies of soil-associated diversity and experimentally testable biosynthetic traits.

## 2. Materials and Methods

### 2.1. Sampling and Isolation

Strains AW10-10^T^ and M8-1^T^ were isolated from forest humus soil collected at an elevation of 1919.47 m in the Gaoligong Mountains (24°49′11.81″ N, 98°44′33.66″ E), Longling County, Yunnan Province, China, whereas strain HGHZ1-2^T^ was isolated from cucumber farmland soil in Mouding County (25°36′23.71″ N, 101°39′53.28″ E), Chuxiong Yi Autonomous Prefecture, Yunnan Province, China. All samples were collected in January 2025.

To obtain culturable bacterial isolates from soil samples collected in Yunnan Province, each sample was serially diluted with sterile distilled water, and 100 μL of the appropriate dilutions was spread onto Luria–Bertani (LB; Tryptone 10 g/L; yeast extract 5 g/L; NaCl 10 g/L; agar 15 g/L) agar plates. After incubation at 28 °C for 5–7 days to recover slow-growing bacteria, colonies with distinct morphological characteristics were selected and purified by repeated streaking on the same medium. Three isolates, designated AW10-10^T^, M8-1^T^ and HGHZ1-2^T^, were obtained and routinely cultivated on LB agar at 37 °C. Purified cultures were preserved at −80 °C in LB broth supplemented with 20% (*v*/*v*) glycerol.

### 2.2. 16S rRNA Gene Sequencing and Phylogenetic Analysis

DNA templates were prepared from fresh bacterial cultures using the heat-lysis method [21]. The nearly complete 16S rRNA gene was amplified using the universal bacterial primers 27F and 1492R [22]. The PCR products were purified and subjected to Sanger sequencing by Tsingke Biotechnology Co., Ltd. (Beijing, China). The resulting forward and reverse sequences were assembled and manually edited using ContigExpress, a component of Vector NTI Suite 6.0 (InforMax, Inc., Bethesda, MD, USA). After assembly, low-quality terminal bases were trimmed, yielding final 16S rRNA gene sequences of 1375–1384 bp. The 16S rRNA gene sequences obtained in this study were submitted to GenBank.

The assembled 16S rRNA gene sequences were compared with those of type strains of species with validly published names using the EzBioCloud database (https://www.ezbiocloud.net/) [23]. Sequences of closely related type strains of the genus *Microbacterium* were retrieved from the GenBank database (https://www.ncbi.nlm.nih.gov/) and aligned using Clustal W implemented in MEGA version 12.0 [24]. Phylogenetic trees were reconstructed using the neighbor-joining (NJ), maximum-likelihood (ML) and maximum-parsimony (MP) methods [25,26,27], with the Kimura two-parameter model applied in both the NJ and ML analyses [28]. The reliability of the tree topologies was evaluated by bootstrap analysis based on 1000 replicates [29].

### 2.3. Genome Sequencing, Phylogenomic and Genomic Analyses

Freshly harvested biomass of strains AW10-10^T^, M8-1^T^, and HGHZ1-2^T^ was submitted to Shanghai Majorbio Bio-pharm Technology Co., Ltd. (Shanghai, China) for shotgun genome sequencing using an Illumina platform. Raw sequencing reads were quality-filtered using fastp version 1.3.3, and the resulting high-quality reads were assembled de novo using SPAdes version 4.2.0 [30,31]. Contigs shorter than 1000 bp were removed from the assemblies using SeqKit version 2.13.0 [32]. Assembly statistics, including N50 values, were calculated using QUAST version 5.3.0, and genome completeness and contamination were assessed using CheckM version 1.2.5 [33,34]. The resulting draft genome assemblies were used for subsequent genome-based and phylogenomic analyses.

The draft genome assemblies of the strains AW10-10^T^, M8-1^T^, and HGHZ1-2^T^ obtained in this study were deposited in GenBank. Based on 16S rRNA gene analysis, genome sequences of closely related *Microbacterium* type strains were retrieved from the NCBI GenBank database. Together with the three newly sequenced genomes, all genomes were annotated using Prokka version 1.15.6 to ensure consistent gene prediction [35]. The resulting predicted amino acid sequences were subjected to orthology analysis using OrthoFinder version 2.5.5 [36]. Single-copy orthologous groups present in all analyzed genomes were retained for phylogenomic reconstruction. For each single-copy orthologous group, the corresponding amino acid sequences were aligned using Clustal Omega version 1.2.4 [37]. The individual alignments were concatenated into a supermatrix, and ambiguously aligned or poorly conserved positions were removed using trimAl version 1.4 [38]. A maximum-likelihood phylogenomic tree was subsequently inferred from the trimmed concatenated alignment using IQ-TREE version 3.1.2 [39]. Branch support was assessed using 1000 ultrafast bootstrap replicates.

For the genome-based relatedness analyses, ANI values between strains AW10-10^T^, M8-1^T^ and HGHZ1-2^T^ and their closely related type strains were calculated using both the OrthoANIu algorithm implemented in the EzBioCloud ANI Calculator and FastANI v1.34 to ensure the robustness of genome-relatedness estimates [40,41]. Digital DNA–DNA hybridization values and their confidence intervals were calculated using the Genome-to-Genome Distance Calculator (GGDC 3.0, https://ggdc.dsmz.de/ggdc.php, accessed on 10 March 2026) based on Formula 2 [42].

As part of the genome-based functional analyses, secondary-metabolite biosynthetic gene clusters, prophage regions and putative antibiotic resistance-associated target genes were predicted using antiSMASH (https://antismash.secondarymetabolites.org/, accessed on 16 May 2026), PHASTEST (https://phastest.ca/, accessed on 16 May 2026) and ARTS (https://arts.ziemertlab.com/, accessed on 16 May 2026), respectively [43,44,45]. All analyses were performed using the default parameters.

### 2.4. Physiological, Biochemical and Chemotaxonomic Characterization

Unless otherwise stated, the strains were cultured on tryptic soy agar (TSA; Tryptone 17 g/L, Soytone 3 g/L, NaCl 5 g/L, agar 15 g/L) plates at 37 °C for 2–3 days. Gram staining was performed using a commercial Gram-staining kit (Solarbio, Beijing, China) according to the manufacturer’s instructions, and cell motility was examined using the hanging-drop method. Cellular morphology was observed by transmission electron microscopy (JEM-2100; JEOL, Tokyo, Japan) after negative staining with 2% (*w*/*v*) phosphotungstic acid. Oxidase activity was determined using the Nadi reaction. Equal volumes of freshly prepared 1% (*w*/*v*) α-naphthol in 95% ethanol and 1% (*w*/*v*) N,N-dimethyl-1,4-phenylenediamine monohydrochloride in distilled water were mixed immediately before use. The development of a blue to blue–purple color within 10 s was considered a positive reaction. Catalase activity was assessed by observing bubble formation following the addition of 5% (*v*/*v*) H_2_O_2_. Growth at different temperatures, pH values and NaCl concentrations was assessed in TSB at 10–50 °C in 5 °C increments, at pH 4.0–12.0 in 1.0-unit increments, and with an additional 0–10% (*w*/*v*) NaCl in 1% increments, respectively. Growth on LB agar, TSA, nutrient agar (NA, Peptone 10 g/L, beef extract 3 g/L, NaCl 5 g/L, agar 15 g/L), International Streptomyces Project medium 2 (ISP 2, yeast extract 4 g/L, malt extract 10 g/L, glucose 4 g/L, agar 15 g/L) and Reasoner’s 2A agar (R2A, yeast extract 0.5 g/L, Peptone 0.5 g/L, casamino acids 0.5 g/L, glucose 0.5 g/L, soluble starch 0.5 g/L, K_2_HPO_4_ 0.3 g/L, MgSO_4_·7H_2_O 0.05 g/L, sodium pyruvate 0.3 g/L, agar 15 g/L) was evaluated. Hydrolysis or degradation of casein, starch, hypoxanthine, xanthine, Tween 20, Tween 80 and chitin was tested on TSA supplemented with the corresponding substrate. Additional physiological and biochemical characteristics were determined using Biolog GEN III MicroPlates (Biolog, Hayward, CA, USA), API 20NE and API ZYM strips (bioMérieux, Marcy-l’Étoile, France), according to the manufacturers’ instructions.

Whole-cell hydrolysates were prepared according to the method of Schleifer and Kandler, and the resulting sugar components were analyzed according to the method of Tang et al. using high-performance liquid chromatography equipped with an Agilent Eclipse XDB-C18 column (4.6 mm × 250 mm, 5 μm; Agilent Technologies, Inc., Santa Clara, CA, USA) [46,47]. Cellular fatty acids were extracted, saponified and methylated according to the method of Sasser [48]. Fatty acid methyl esters were analyzed using an Agilent 7890 gas chromatograph equipped with the Sherlock Microbial Identification System version 6.0 and identified using the standard MIS Library TSBA6. Respiratory quinones were extracted and analyzed according to the method of Tamaoka et al. [49]. Polar lipids were extracted and analyzed according to the method of Minnikin et al. and separated by two-dimensional thin-layer chromatography [50]. Phosphomolybdic acid, ninhydrin, molybdenum blue and α-naphthol reagents were used to detect total lipids, aminolipids, phospholipids and glycolipids, respectively.

## 3. Results

### 3.1. 16S rRNA Gene Phylogeny

The quality-trimmed 16S rRNA gene sequences of strains AW10-10^T^, M8-1^T^ and HGHZ1-2^T^ were 1375, 1379 and 1384 bp in length, respectively, and were deposited in GenBank under accession numbers PZ263861, PZ263859 and PZ263862, respectively. Pairwise comparison showed that the three strains shared 16S rRNA gene sequence similarities of AW10-10^T^ versus M8-1^T^ 96.2%, AW10-10^T^ versus HGHZ1-2^T^ 96.3% and M8-1^T^ versus HGHZ1-2^T^ 97.9%.

EzBioCloud analysis indicated that strain AW10-10^T^ showed the highest 16S rRNA gene sequence similarities to *Microbacterium ulmi* XIL02^T^ (97.3%), *M. protaetiae* DFW100M-13^T^ (97.2%) and *M. immunditiarum* SK 18^T^ (97.2%). Strain M8-1^T^ was most closely related to *M. azadirachtae* AI-S262^T^ (98.6%), *M. natoriense* TNJL143-2^T^ (98.3%) and *M. resistens* NBRC 103078^T^ (98.3%). The closest relatives of strain HGHZ1-2^T^ were *M. pullorum* Sa4CUA7^T^ (98.6%), *M. resistens* NBRC 103078^T^ (98.5%) and *M. natoriense* TNJL143-2^T^ (98.5%). The remaining type strains of species of the genus *Microbacterium* exhibited sequence similarities below 97.2%, 98.3% and 98.5% to strains AW10-10^T^, M8-1^T^ and HGHZ1-2^T^, respectively.

**Figure 1:**
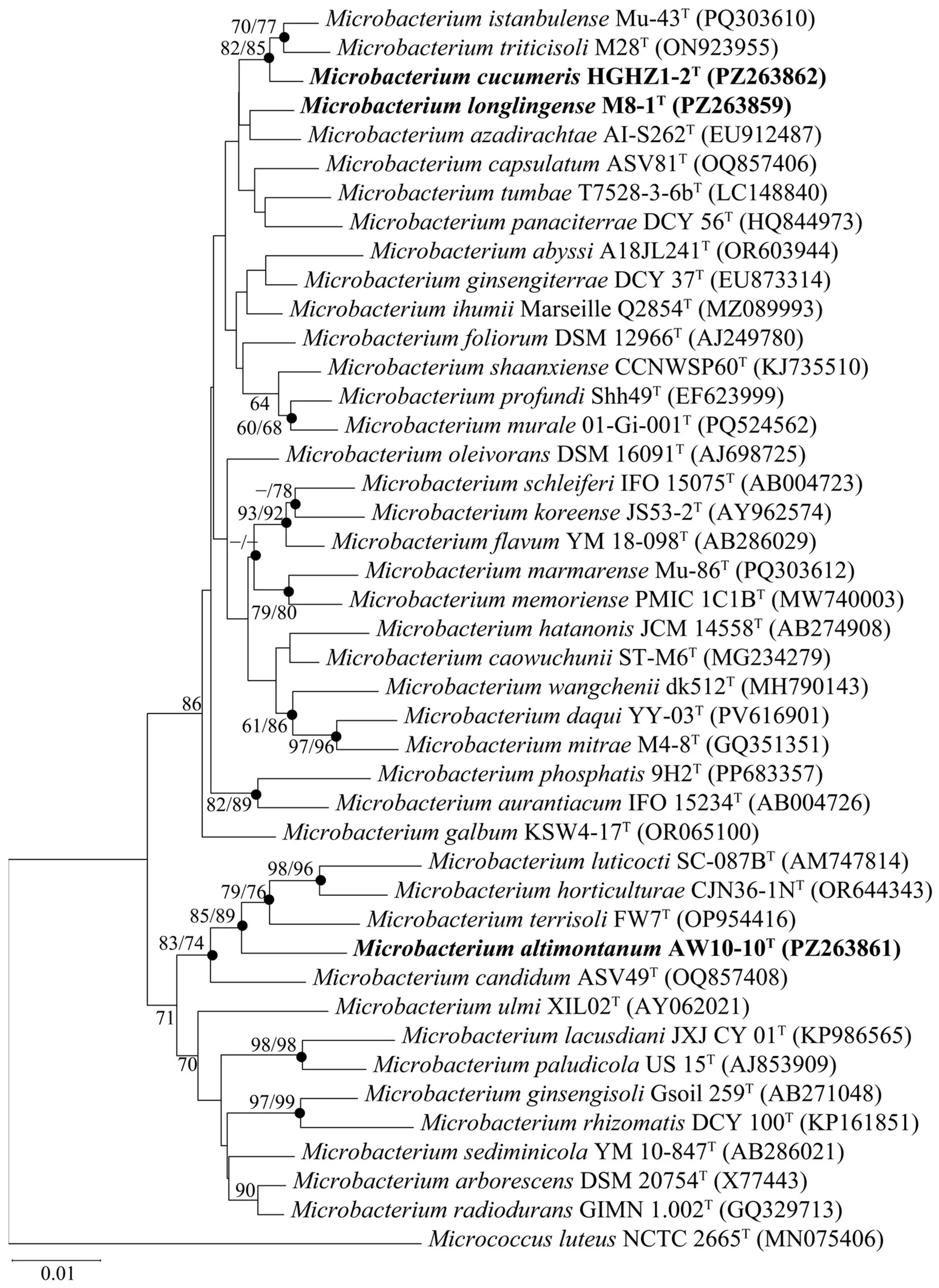
NJ phylogenetic tree based on 16S rRNA gene sequences showing the relationships of strains AW10-10^T^, M8-1^T^ and HGHZ1-2^T^ with closely related members of the genus *Microbacterium*. Numbers at nodes represent bootstrap percentages obtained from the NJ and ML analyses, respectively, based on 1000 replicates. Paired values separated by a slash are presented in the order NJ/ML. A single value indicates that the corresponding node was supported only by the NJ analysis, whereas the node was not recovered in the ML analysis. Filled circles (·) indicate nodes recovered in both NJ and ML analyses. Only bootstrap values of 60% or higher are shown. *Micrococcus luteus* NCTC 2665^T^ was used as the outgroup. Bar, 0.01 substitutions per nucleotide position.

In the neighbor-joining and maximum-likelihood phylogenetic trees, strains AW10-10^T^, M8-1^T^ and HGHZ1-2^T^ were consistently placed within the genus *Microbacterium* (Figure 1 and Figure S1). Strain AW10-10^T^ formed a cluster with *M. luticocti* SC-087B^T^, *M. horticulturae* CJN36-1N^T^, *M. terrisoli* FW7^T^, and *M. candidum* ASV49^T^, whereas strains M8-1^T^ and HGHZ1-2^T^ were most closely associated with *M. istanbulense* Mu-43^T^, *M. triticisoli* M28^T^, and *M. azadirachtae* AI-S262^T^. The three strains occupied separate phylogenetic positions and did not form a single coherent lineage. The maximum-parsimony tree recovered broadly congruent relationships, although minor differences were observed in the arrangement of some neighboring branches (Figure S2).

### 3.2. Genome Characteristics, Phylogenomic Analysis and Genome Relatedness

The draft genome assemblies of strains AW10-10^T^, M8-1^T^ and HGHZ1-2^T^ comprised 3,587,685, 3,273,084 and 3,544,967 bp, respectively. The assemblies consisted of 16, 19 and 11 contigs, with N50 values of 450,853 bp, 311,765 bp and 545,534 bp, respectively. The three genomes were sequenced to average depths of approximately 279×, 306×, and 282×, respectively. Genome completeness values were estimated at 99.5%, 100.0% and 99.5% for strains AW10-10^T^, M8-1^T^ and HGHZ1-2^T^, respectively, with corresponding contamination values of 0.5%, 0.8% and 0.5%. These results indicated that the draft genome assemblies were of sufficient quality for genome-based taxonomic analyses. Based on the draft genome assemblies, the DNA G + C contents of strains AW10-10^T^, M8-1^T^ and HGHZ1-2^T^ were 70.0, 69.7 and 69.5 mol%, respectively. Genome annotation identified 3397 coding sequences (CDSs), 87 tRNA genes and three rRNA genes in strain AW10-10^T^; 3064 CDSs, 48 tRNA genes and four rRNA genes in strain M8-1^T^; and 3314 CDSs, 50 tRNA genes and three rRNA genes in strain HGHZ1-2^T^. The relatively low numbers of rRNA genes recovered from the draft genome assemblies most likely reflect the difficulty of resolving highly conserved and repetitive rRNA operons using short-read sequencing, which can result in repeat collapse or fragmentation at contig boundaries. Therefore, the annotated rRNA gene counts should not be interpreted as the actual rRNA operon copy numbers. The draft genome sequences of strains AW10-10^T^, M8-1^T^ and HGHZ1-2^T^ were deposited in GenBank under accession numbers JBYRJB000000000, JBYRIZ000000000 and JBYRJA000000000, respectively.

**Figure 2:**
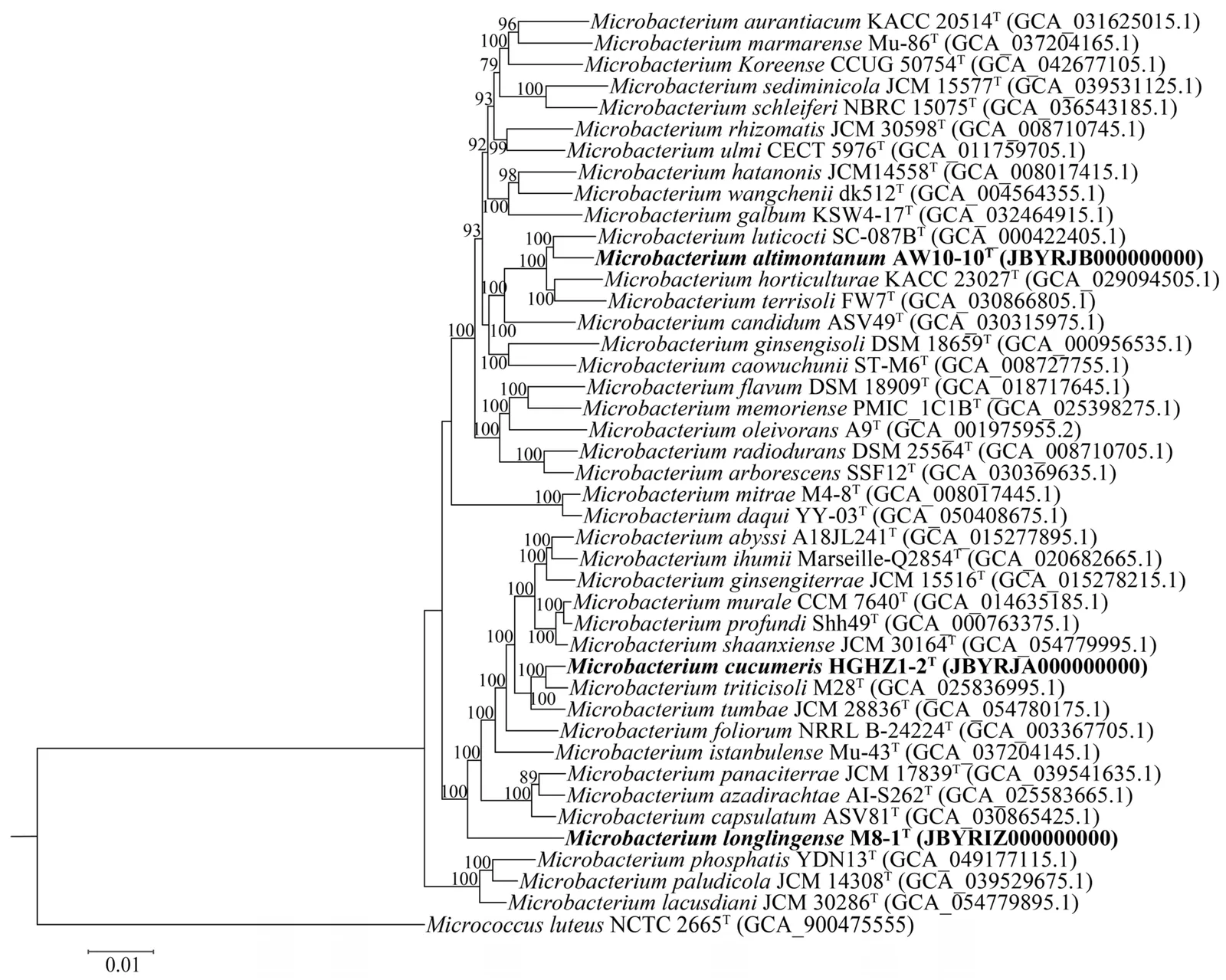
Maximum-likelihood phylogenomic tree showing the relationships of strains AW10-10^T^, M8-1^T^ and HGHZ1-2^T^ with closely related members of the genus *Microbacterium*. The tree was reconstructed based on the concatenated amino acid sequences of 522 single-copy core genes. Numbers at nodes indicate ultrafast bootstrap percentages based on 1000 replicates; only values of 70% or higher are shown. *Micrococcus luteus* NCTC 2665^T^ was used as the outgroup. Bar, 0.01 substitutions per amino acid position.

The maximum-likelihood phylogenomic tree reconstructed from the concatenated amino acid sequences of 522 single-copy core genes was rooted using *Micrococcus luteus* NCTC 2665^T^ as the outgroup (Figure 2). Strain AW10-10^T^ was sister to *M. luticocti* DSM 19459^T^ with 100% ultrafast bootstrap support. The clade comprising these two strains was further resolved as sister to the clade formed by *M. horticulturae* KACC 23027^T^ and *M. terrisoli* FW7^T^, also with 100% ultrafast bootstrap support. Strain M8-1^T^ formed a distinct lineage that was sister to a clade comprising *M. istanbulense* Mu-43^T^, *M. panaciterrae* JCM 17839^T^, *M. azadirachtae* CNUC13^T^ and *M. capsulatum* ASV81^T^, with 100% ultrafast bootstrap support. Strain HGHZ1-2^T^ was sister to *M. triticisoli* M28^T^, and the clade comprising these two strains was further grouped with *M. tumbae* JCM 28836^T^; both nodes received 100% ultrafast bootstrap support. Overall, the three strains occupied separate and strongly supported positions in the phylogenomic tree, indicating their distinct phylogenetic placements within the genus *Microbacterium*.

The OrthoANI, FastANI and dDDH values calculated from the genome assemblies between strains AW10-10^T^, M8-1^T^ and HGHZ1-2^T^ and their closely related type strains ranged from 75.7 to 79.6%, from 79.9 to 81.9% and from 19.7 to 22.5%, respectively. Pairwise comparisons among the three strains based on the genome assemblies yielded OrthoANI values of 75.0–76.4%, FastANI values of 79.1–79.8% and dDDH values of 20.1–21.5% (Table S1). The relatively high 16S rRNA gene sequence similarities, reaching approximately 98.6%, contrasted with the substantially lower genome-wide ANI values. This discordance most likely reflects the highly conserved nature of the 16S rRNA gene, which represents only a small fraction of the bacterial genome and may therefore fail to capture extensive divergence across the remainder of the genome. Similar discordance has also been observed among previously described species of the genus *Microbacterium*. For example, *M. memoriense* and *M. marmarense* shared approximately 98.6% 16S rRNA gene sequence similarity but showed an OrthoANI value of approximately 73.0%; *M. triticisoli* and *M. thalassium* shared approximately 98.6% 16S rRNA gene sequence similarity but had an OrthoANI value of 75.9%; and *M. pullorum* and *M. galbinum* shared approximately 98.7% 16S rRNA gene sequence similarity but showed an OrthoANI value of only 75.6%. These examples indicate that relatively high 16S rRNA gene similarity accompanied by OrthoANI values below 76% is not unique within the genus *Microbacterium*. These observations further support the use of genome-wide indices, together with phylogenetic and phenotypic evidence, for reliable species delineation within the genus *Microbacterium*. All ANI and dDDH values obtained from comparisons involving the three strains were far below the generally accepted species delineation thresholds of 95–96% and 70%, respectively, supporting the recognition of strains AW10-10^T^, M8-1^T^ and HGHZ1-2^T^ as representatives of three distinct novel species within the genus *Microbacterium*.

### 3.3. Genome Mining

Genome mining revealed strain-specific differences in predicted secondary-metabolite biosynthetic gene clusters, prophage regions and putative antimicrobial resistance-associated genes or protein models.

The antiSMASH analysis identified eight putative biosynthetic gene clusters (BGCs) in strain AW10-10^T^ and three BGCs each in strains M8-1^T^ and HGHZ1-2^T^ (Table S2). Strain AW10-10^T^ possessed the largest predicted BGC repertoire, comprising clusters classified as phenazine, terpene, type III polyketide synthase (T3PKS), terpene-precursor, β-lactone and RiPP-like types. The terpene clusters showed similarity to carotenoid-related BGCs; bacterial carotenoid pigments can contribute to pigmentation and protection against oxidative stress [51]. Although phenazine antibiotics have been characterized in *Pseudomonas* species [52], the phenazine-related hit detected in strain AW10-10^T^ showed only low-confidence similarity and was biologically unexpected for a member of the genus *Microbacterium*. Therefore, this prediction was not interpreted as evidence of a functional phenazine biosynthetic pathway. Strain M8-1^T^ contained a cluster showing high-confidence similarity to the ε-poly-L-lysine biosynthetic cluster, suggesting putative genetic potential for ε-poly-L-lysine biosynthesis [53]. In strain HGHZ1-2^T^, a hybrid T3PKS/β-lactone/terpene-precursor cluster showed low similarity to the vibriobactin biosynthetic cluster [54]; however, this weak similarity was insufficient to infer siderophore production or iron-acquisition activity. Several additional clusters showed no identifiable similarity to characterized BGCs. Overall, these predictions indicate only putative biosynthetic potential, and the production, chemical identities and biological functions of the corresponding metabolites require experimental validation.

Beyond the predicted biosynthetic potential, genome-based PHASTEST analysis identified a single putatively intact prophage region in the draft genome assembly of strain AW10-10^T^, whereas no prophage regions were predicted in strains M8-1^T^ or HGHZ1-2^T^. The predicted region was located on contig 4 at positions 270,575–312,959 bp, spanned approximately 42.4 kb and encoded 63 proteins, with a completeness score of 150 (where PHASTEST scores range from 0 to 150, with 150 indicating a fully intact prophage) (Table S3). Nine of the predicted proteins showed their closest matches to proteins of Microbacterium phage Min1 (NC_009603), indicating partial similarity to this phage. The DNA G + C content of the prophage region was 67.7 mol%, which was 2.24 percentage points lower than the genome-wide value of strain AW10-10^T^ (70.0 mol%). Prophages can contribute to bacterial genome plasticity and influence host functions through horizontal gene transfer or regulatory interactions [55,56]. Nevertheless, classification of the region as intact by PHASTEST does not demonstrate that it is inducible or capable of producing infectious particles, and experimental verification is required.

Finally, ARTS analysis predicted several putative antimicrobial resistance-associated genes or protein models in the draft genome assemblies of strains AW10-10^T^, M8-1^T^ and HGHZ1-2^T^, with their identities, numbers and strain-specific distributions summarized in Table S4. Some predicted models, including DNA gyrase B and DNA topoisomerase IV, represent conserved housekeeping proteins and common antibacterial targets and were therefore not interpreted as evidence of antimicrobial resistance [57]. Likewise, detection of a vanS-like model alone does not establish the presence of a complete VanS/VanR regulatory system or a functional vancomycin-resistance pathway [58]. Overall, ARTS provides sequence-based predictions of putative resistance-associated genes or protein models rather than evidence of phenotypic antimicrobial resistance. Because antimicrobial susceptibility testing was not performed in this study, no conclusions were drawn regarding the antimicrobial susceptibility or resistance phenotypes of the three strains.

### 3.4. Phenotypic and Chemotaxonomic Characteristics

All three strains are Gram-positive, aerobic, and rod-shaped bacteria (Figure S3). Strains AW10-10^T^ and HGHZ1-2^T^ were motile, whereas strain M8-1^T^ was non-motile. Cells of strains AW10-10^T^, M8-1^T^ and HGHZ1-2^T^ measured 0.3–0.6 × 0.6–1.3 μm, 0.3–0.6 × 0.6–1.0 μm and 0.2–0.4 × 0.8–1.3 μm, respectively. Catalase activity tests showed all three strains were positive. Oxidase activity tests showed the strain AW10-10^T^ was positive, while strains M8-1^T^ and HGHZ1-2^T^ were negative. Strain AW10-10^T^ grew at 10–45 °C, at pH 5.0–8.0 and in the presence of 0–4% (*w*/*v*) NaCl. Strains M8-1^T^ and HGHZ1-2^T^ grew at 10–45 °C, at pH 6.0–10.0 and in the presence of 0–8% (*w*/*v*) NaCl. Optimal growth of all three strains occurred at 35–40 °C, at pH 7.0, and in the absence of added NaCl. All three strains grew best on TSA medium, and their growth on the five media is shown in Table 1. Detailed physiological and biochemical characteristics determined using the API 20NE, API ZYM and Biolog GEN III systems are presented in Table S5. In addition, the three strains differed from one another in motility, growth pH range, NaCl tolerance and several enzyme activities and substrate-utilization patterns. The principal differential physiological and biochemical characteristics of strains AW10-10^T^, M8-1^T^ and HGHZ1-2^T^ and their closely related type strains identified in the phylogenetic analyses are presented in Table 2.

Whole-cell hydrolysates of strains AW10-10^T^, M8-1^T^ and HGHZ1-2^T^ contained ribose, galactose, mannose and glucose. Small amounts of rhamnose were additionally detected in strains AW10-10^T^ and M8-1^T^ but not in strain HGHZ1-2^T^. The cellular fatty acid profiles of all three strains were dominated by anteiso-C_15:0_, anteiso-C_17:0_ and iso-C_16:0_, though their relative proportions varied among the strains. In strains AW10-10^T^ and HGHZ1-2^T^, anteiso-C_17:0_ was the most abundant, followed by anteiso-C_15:0_ and iso-C_16:0_, whereas in strain M8-1^T^, anteiso-C_15:0_ was the most abundant, followed by anteiso-C_17:0_ and iso-C_16:0_ (Table S6). Respiratory quinone analysis showed that the major menaquinones of strain AW10-10^T^ were MK-12 (68.0%) and MK-13 (32.0%). The major menaquinones of strain M8-1^T^ were MK-10 (39.9%), MK-11 (33.3%) and MK-9 (18.6%), with MK-12 (8.3%) detected as a minor component. The major menaquinones of strain HGHZ1-2^T^ were MK-13 (72.9%) and MK-12 (27.1%). The polar lipid profiles of strains AW10-10^T^ and HGHZ1-2^T^ consisted of diphosphatidylglycerol (DPG), phosphatidylglycerol (PG) and one unidentified glycolipid (GL), whereas that of strain M8-1^T^ comprised DPG, PG and two unidentified glycolipids (GLs) (Figure S4). These chemotaxonomic characteristics supported the assignment of the three strains to the genus *Microbacterium*, while differences in their fatty acid proportions, menaquinone profiles and polar lipid compositions provided additional characteristics for distinguishing them from one another and from their closely related type strains.

## 4. Conclusions

A polyphasic taxonomic approach combining phenotypic, chemotaxonomic, phylogenetic and genome-based evidence demonstrated that strains AW10-10^T^, M8-1^T^ and HGHZ1-2^T^ represent three distinct species of the genus *Microbacterium*. The three strains formed separate phylogenetic lineages, and their ANI and dDDH values relative to closely related type strains were well below the generally accepted species delineation thresholds. Their physiological, biochemical and chemotaxonomic characteristics were consistent with those of the genus *Microbacterium*, while differences in growth ranges, biochemical reactions, cellular fatty acid profiles, menaquinone compositions, whole-cell sugars and polar lipid patterns enabled them to be distinguished from their closest relatives. Accordingly, the names *Microbacterium altimontanum* sp. nov., *Microbacterium longlingense* sp. nov. and *Microbacterium cucumeris* sp. nov. are proposed for strains AW10-10^T^, M8-1^T^ and HGHZ1-2^T^, respectively.

Genome-mining analyses revealed several strain-specific predicted genomic features. Strain AW10-10^T^ possessed the largest predicted repertoire of biosynthetic gene clusters and harbored a putatively intact prophage region, whereas strain M8-1^T^ contained a high-confidence antiSMASH match to an ε-poly-L-lysine biosynthetic cluster, suggesting potential genetic capacity for its biosynthesis. Strain HGHZ1-2^T^ contained a hybrid biosynthetic gene cluster showing only low similarity to the vibriobactin biosynthetic cluster. However, low-confidence matches, particularly the phenazine-related hit in strain AW10-10^T^ and the vibriobactin-related hit in strain HGHZ1-2^T^, do not demonstrate the presence of functional biosynthetic pathways or the production of the corresponding metabolites and therefore require experimental validation.

ARTS analysis also identified several putative antimicrobial resistance-associated genes or protein models in the three genome assemblies. However, some predictions corresponded to conserved housekeeping proteins or common antibacterial targets, and ARTS predictions alone do not provide evidence of phenotypic antimicrobial resistance. A limitation of the present study is that antimicrobial susceptibility testing was not performed. Consequently, no conclusions can be drawn regarding the antimicrobial resistance phenotypes of the three strains, and the genomic predictions require validation through standardized antimicrobial susceptibility testing and functional analyses. Overall, the characterization of these strains broadens the taxonomic and genomic representation of soil-derived members of the genus *Microbacterium* and provides reference strains and genome sequences for future comparative studies and experimental validation of their predicted genomic features.

### 4.1. Description of Microbacterium altimontanum sp. nov.

*Microbacterium altimontanum* (al.ti.mon.ta′num. N.L. neut. adj. *altimontanum*, pertaining to a high mountain, referring to the mountainous locality in the Gaoligong Mountains, Yunnan Province, China, where the type strain was isolated).

Cells are Gram-stain-positive, aerobic, motile and rod-shaped (0.3–0.6 × 0.6–1.3 μm). Cells are catalase-positive and oxidase-positive. Colonies on TSA are yellow, circular, smooth and convex. Growth occurs at 10–45 °C (optimum, 35–40 °C), at pH 5.0–8.0 (optimum, pH 7.0) and with 0–4% (*w*/*v*) NaCl (optimum in the absence of added NaCl). Aesculin is hydrolysed, but casein, starch, xanthine, hypoxanthine, Tweens 20 and 80, gelatin and chitin are not hydrolysed.

In the API 20NE system, positive results are obtained for nitrate reduction, aesculin hydrolysis and β-galactosidase activity (PNPG), and D-mannose and D-mannitol are assimilated. Negative results are obtained for indole production, glucose fermentation, arginine dihydrolase, urease and gelatin hydrolysis, and for the assimilation of D-glucose, L-arabinose, N-acetyl-D-glucosamine, maltose, potassium gluconate, capric acid, adipic acid, malic acid, citrate and phenylacetic acid. In the API ZYM system, positive reactions are observed for esterase (C4), esterase lipase (C8), leucine arylamidase, valine arylamidase, cystine arylamidase, trypsin, acid phosphatase, naphthol-AS-BI-phosphohydrolase, α-glucosidase, β-glucosidase, N-acetyl-β-glucosaminidase and α-mannosidase. Negative reactions are observed for alkaline phosphatase, lipase (C14), α-chymotrypsin, α-galactosidase, β-galactosidase, β-glucuronidase and α-fucosidase.

The whole-cell sugars are ribose, galactose, mannose and glucose, together with a small amount of rhamnose. The major cellular fatty acids are anteiso-C_17:0_, anteiso-C_15:0_ and iso-C_16:0_. The predominant menaquinones are MK-12 and MK-13. The polar lipids comprise DPG, PG and one unidentified glycolipid. The DNA G + C content of the type-strain genome is 70.0 mol%.

The type strain, AW10-10^T^ (=GDMCC 16749^T^=KCTC 59693^T^), was isolated from forest humus soil collected at an elevation of 1919.47 m in the Gaoligong Mountains, Longling County, Yunnan Province, China (24°49′11.81″ N, 98°44′33.66″ E). The GenBank accession numbers for the 16S rRNA gene and draft genome sequences are PZ263861 and JBYRJB000000000, respectively.

### 4.2. Description of Microbacterium longlingense sp. nov.

*Microbacterium longlingense* (long.ling.en’se. N.L. neut. adj. *longlingense*, pertaining to Longling County, Yunnan Province, China, where the type strain was isolated).

Cells are Gram-stain-positive, aerobic, non-motile and rod-shaped (0.3–0.6 × 0.6–1.0 μm). Cells are catalase-positive and oxidase-negative. Colonies on TSA are white, circular, smooth and convex. Growth occurs at 10–45 °C (optimum, 35–40 °C), at pH 6.0–10.0 (optimum, pH 7.0) and with 0–8% (*w*/*v*) NaCl (optimum in the absence of added NaCl). Aesculin is hydrolysed, but casein, starch, xanthine, hypoxanthine, Tweens 20 and 80, gelatin and chitin are not hydrolysed.

In the API 20NE system, positive results are obtained for arginine dihydrolase, aesculin hydrolysis and β-galactosidase activity (PNPG), and D-glucose, D-mannose, D-mannitol, N-acetyl-D-glucosamine, maltose, potassium gluconate, malic acid and citrate are assimilated. Negative results are obtained for nitrate reduction, indole production, glucose fermentation, urease and gelatin hydrolysis, and for the assimilation of L-arabinose, capric acid, adipic acid and phenylacetic acid. In the API ZYM system, positive reactions are observed for esterase (C4), esterase lipase (C8), leucine arylamidase, valine arylamidase, cystine arylamidase, trypsin, acid phosphatase, naphthol-AS-BI-phosphohydrolase, α-glucosidase, β-glucosidase, N-acetyl-β-glucosaminidase and α-mannosidase. Negative reactions are observed for alkaline phosphatase, lipase (C14), α-chymotrypsin, α-galactosidase, β-galactosidase, β-glucuronidase and α-fucosidase.

The whole-cell sugars are ribose, galactose, mannose and glucose, together with a small amount of rhamnose. The major cellular fatty acids are anteiso-C_15:0_, anteiso-C_17:0_ and iso-C_16:0_. The major menaquinones are MK-10, MK-11 and MK-9, with MK-12 present as a minor component. The polar lipids comprise DPG, PG and two unidentified glycolipids. The DNA G + C content of the type-strain genome is 69.7 mol%.

The type strain, M8-1^T^ (=GDMCC 16752^T^=KCTC 59696^T^), was isolated from forest humus soil collected at an elevation of 1919.47 m in the Gaoligong Mountains, Longling County, Yunnan Province, China (24°49′11.81″ N, 98°44′33.66″ E). The GenBank accession numbers for the 16S rRNA gene and draft genome sequences are PZ263859 and JBYRIZ000000000, respectively.

### 4.3. Description of Microbacterium cucumeris sp. nov.

*Microbacterium cucumeris* (cu.cu’me.ris. L. gen. masc. n. *cucumeris*, of a cucumber, referring to the cucumber-associated source from which the type strain was isolated).

Cells are Gram-stain-positive, aerobic, motile and rod-shaped (0.2–0.4 × 0.8–1.3 μm). Cells are catalase-positive and oxidase-negative. Colonies on TSA are white, circular, smooth and convex. Growth occurs at 10–45 °C (optimum, 35–40 °C), at pH 6.0–10.0 (optimum, pH 7.0) and with 0–8% (*w*/*v*) NaCl (optimum in the absence of added NaCl). Aesculin and gelatin are hydrolysed, but casein, starch, xanthine, hypoxanthine, Tweens 20 and 80 and chitin are not hydrolysed.

In the API 20NE system, positive results are obtained for nitrate reduction, aesculin and gelatin hydrolysis and β-galactosidase activity (PNPG), and D-glucose, D-mannose, D-mannitol and maltose are assimilated. Negative results are obtained for indole production, glucose fermentation, arginine dihydrolase and urease, and for the assimilation of L-arabinose, N-acetyl-D-glucosamine, potassium gluconate, capric acid, adipic acid, malic acid, citrate and phenylacetic acid. In the API ZYM system, positive reactions are observed for esterase (C4), esterase lipase (C8), leucine arylamidase, valine arylamidase, cystine arylamidase, trypsin, acid phosphatase, naphthol-AS-BI-phosphohydrolase, α-galactosidase, β-galactosidase, α-glucosidase, β-glucosidase, N-acetyl-β-glucosaminidase and α-fucosidase. Negative reactions are observed for alkaline phosphatase, lipase (C14), α-chymotrypsin, β-glucuronidase and α-mannosidase.

The whole-cell sugars are ribose, galactose, mannose and glucose. The major cellular fatty acids are anteiso-C_17:0_, anteiso-C_15:0_ and iso-C_16:0_. The predominant menaquinones are MK-13 and MK-12. The polar lipids comprise DPG, PG and one unidentified glycolipid. The DNA G + C content of the type-strain genome is 69.5 mol%.

The type strain, HGHZ1-2^T^ (=GDMCC 16751^T^=KCTC 59694^T^), was isolated in January 2025 from soil collected from a cucumber field in Mouding County, Chuxiong Yi Autonomous Prefecture, Yunnan Province, China (25°36′23.71″ N, 101°39′53.28″ E). The GenBank accession numbers for the 16S rRNA gene and draft genome sequences are PZ263862 and JBYRJA000000000, respectively.

## Figures and Tables

**Table 1:** Growth of the three strains on different media. Growth rate: +++, fastest (optimal medium); ++, positive but slower; +, microcolonies only; −, no growth. Strain color: observed colony color; −: not determined (no growth or colonies too small).

Culture Medium	AW10-10^T^		M8-1^T^		HGHZ1-2^T^	
	Growth Rate	Strain Color	Growth Rate	Strain Color	Growth Rate	Strain Color
LB	++	Yellow	++	White	++	Light yellow
NA	+	−	++	White	++	Light yellow
ISP2	+	−	−	−	++	Light yellow
R2A	+	−	++	White	+	−
TSA	+++	Yellow	+++	White	+++	White

**Table 2:** Differential phenotypic and chemotaxonomic characteristics of strains AW10-10^T^, M8-1^T^ and HGHZ1-2^T^ and selected closely related type strains. Strains: 1, AW10-10^T^; 2, *M. luticocti* SC-087B^T^ [59]; 3, *M. horticulturae* CJN36-1N^T^ [8]; 4, *M. terrisoli* FW7^T^ [60]; 5, *M. candidum* ASV49^T^ [10]; 6, M8-1^T^; 7, HGHZ1-2^T^; 8, *M. istanbulense* Mu-43^T^ [61]; 9, *M. triticisoli* M28^T^ [15]; 10, *M. azadirachtae* AI-S262^T^ [7]. +: positive, −: negative, W: weak, ND: not determined.

Characteristics	1	2	3	4	5	6	7	8	9	10
Temperature range for growth (°C)	10–45	25–45	10–37	10–37	4–37	10–45	10–45	15–42	15–38	10–37
Motility	+	+	−	−	−	−	+	−	−	+
NaCl tolerance for growth (% *w*/*v*)	0–4	0–10	0–7	0–5	0–1	0–8	0–8	0–1	0–8	0–5.5
pH range for growth	5–8	5.5–10	5–8	6–10	6–9	6–10	6–10	ND	5–8.5	5–10
Catalase	+	−	−	+	+	+	+	−	+	+
Oxidase	+	−	ND	−	−	−	−	+	−	−
Nitrate reduction	+	+	+	+	−	−	+	−	+	−
Hydrolysis of:										
Casein	−	ND	−	−	+	−	−	−	−	+
Starch	−	W	−	+	+	−	−	−	−	−
Tween 20	−	ND	ND	ND	ND	−	−	+	ND	ND
Gelatin	−	+	+	+	−	−	+	−	+	−
Aesculin	+	+	+	+	ND	+	+	−	ND	+
Assimilation of:										
L-Alanine	W	ND	−	−	ND	+	+	−	−	+
L-Arabinose	−	−	+	+	+	−	−	−	+	+
Citrate	−	−	+	−	ND	+	−	−	−	+
D-Malate	−	−	+	−	ND	+	−	−	−	+
Menaquinones (MK)	12;13	11;12	10;11;12	11;12;13	11;12	9;10;11;12	12;13	ND	10;11;12	12;13
Major polar lipids	DPG, PG, GL	DPG, PG, GL, PL	DPG, PG, 3ALs, 2GLs, L	DPG, PG, 2ALs	DPG, PG, 2PLs	DPG, PG, 2GLs	DPG, PG, GL	ND	DPG, PG, GL	ND
G + C content (mol%)	70.0	72.0	68.5	68.7	69.5	69.7	69.5	68.4	68.8	69.5

## Data Availability

The original data presented in the study are openly available in NCBI GenBank database at PZ263861, PZ263859, PZ263862, JBYRJB000000000, JBYRIZ000000000 and JBYRJA000000000.

## Supplementary Materials

The following supporting information can be downloaded at: https://www.mdpi.com/article/10.3390/microorganisms14081644/s1, Figure S1: Maximum-likelihood phylogenetic tree based on 16S rRNA gene sequences showing the relationships of strains AW10-10^T^, M8-1^T^ and HGHZ1-2^T^ with members of the genus *Microbacterium*. Bootstrap values based on 1000 replicates are shown at branch nodes when greater than 60%. *Micrococcus luteus* NCTC 2665^T^ was used as the outgroup. The scale bar represents 0.01 substitutions per nucleotide position; Figure S2: Maximum-parsimony phylogenetic tree based on 16S rRNA gene sequences showing the relationships of strains AW10-10^T^, M8-1^T^ and HGHZ1-2^T^ with members of the genus *Microbacterium*. Bootstrap values based on 1000 replicates are shown at branch nodes when greater than 60%. *Micrococcus luteus* NCTC 2665^T^ was used as the outgroup. The scale bar represents 20 nucleotide substitutions; Figure S3: Transmission electron micrographs of strains AW10-10^T^, M8-1^T^ and HGHZ1-2^T^. Scale bars: 500 nm for AW10-10^T^ and M8-1^T^, and 200 nm for HGHZ1-2^T^; Figure S4: Two-dimensional thin-layer chromatograms of the polar lipids of strains AW10-10^T^, M8-1^T^, and HGHZ1-2^T^; Table S1: 16S rRNA gene sequence similarities, average nucleotide identity (ANI) and digital DNA–DNA hybridization (dDDH) values among strains AW10-10^T^, M8-1^T^ and HGHZ1-2^T^ and their five closest type strains; Table S2: Secondary metabolite biosynthetic gene clusters identified in strains AW10-10^T^, M8-1^T^, and HGHZ1-2^T^ using the antiSMASH; Table S3: A prophage region was identified in the genome of strain AW10-10^T^ using PHASTEST; Table S4: Antimicrobial resistance models were detected in the genomes of strains AW10-10^T^, M8-1^T^, and HGHZ1-2^T^ using the ARTS server; Table S5: Phenotypic characteristics of strains AW10-10^T^, M8-1^T^, and HGHZ1-2^T^ determined using Biolog GENIII, API 20NE, and API ZYM systems; Table S6: Cellular fatty acid composition (%) of strains AW10-10^T^, M8-1^T^, and HGHZ1-2^T^ and their related type strains of the genus *Microbacterium*.

## Abbreviations

The following abbreviations are used in this manuscript:

dDDH	Digital DNA-DNA hybridization
ANI	Average nucleotide identity
LB	Luria–Bertani
TSA	Tryptic soy agar
R2A	Reasoner’s 2A
NA	Nutrient agar
ISP2	International Streptomyces Project 2
NJ	Neighbor-joining
ML	Maximum-likelihood
MP	Maximum-parsimony
BGCs	Biosynthetic gene clusters

## References

[1] Orla-Jensen S. (1919). The Lactic Acid Bacteria.

[2] Takeuchi M., Hatano K. (1998). Union of the genera *Microbacterium* Orla-Jensen and *Aureobacterium* Collins et al. in a redefined genus *Microbacterium*. Int. J. Syst. Bacteriol..

[3] Fidalgo C., Riesco R., Henriques I., Trujillo M.E., Alves A. (2016). *Microbacterium diaminobutyricum* sp. nov., isolated from *Halimione portulacoides*, which contains diaminobutyric acid in its cell wall, and emended description of the genus *Microbacterium*. Int. J. Syst. Evol. Microbiol..

[4] Goodfellow M., Kämpfer P., Busse H.-J., Trujillo M.E., Suzuki K.-I., Ludwig W., Whitman W.B. (2012). Bergey’s Manual of Systematic Bacteriology: The Actinobacteria.

[5] Evtushenko L.I., Takeuchi M. (2006). The family Microbacteriaceae. The Prokaryotes.

[6] Lee S.D., Yang H.L., Kim I.S. (2023). Four new *Microbacterium* species isolated from seaweeds and reclassification of five *Microbacterium* species with a proposal of *Paramicrobacterium* gen. nov. under a genome-based framework of the genus *Microbacterium*. Front. Microbiol..

[7] Madhaiyan M., Poonguzhali S., Lee J.-S., Lee K.-C., Saravanan V.S., Santhanakrishnan P. (2010). *Microbacterium azadirachtae* sp. nov., a plant-growth-promoting actinobacterium isolated from the rhizoplane of neem seedlings. Int. J. Syst. Evol. Microbiol..

[8] Choi H., Choi Y., Kim S., Kim Y., Naito H., Yamada T., Hamada M., Kim N., Lee Y., Heo J. (2024). *Microbacterium horticulturae* sp. nov., a novel actinobacterium isolated from flowerpot soil. Int. J. Syst. Evol. Microbiol..

[9] Ding Y., Ding X., Chen Y., Wei S., Zhang G. (2024). *Microbacterium abyssi* sp. nov. and *Microbacterium limosum* sp. nov., two new species of the genus *Microbacterium*, isolated from deep-sea sediment samples. Int. J. Syst. Evol. Microbiol..

[10] Wen W., Zhao Y., Xie B., Lv J. (2025). *Microbacterium candidum* sp. nov. and *Microbacterium capsulatum* sp. nov., isolated from waste landfill. Int. J. Syst. Evol. Microbiol..

[11] Cordovez V., Schop S., Hordijk K., Dupré de Boulois H., Coppens F., Hanssen I., Raaijmakers J.M., Carrión V.J. (2018). Priming of plant growth promotion by volatiles of root-associated *Microbacterium* spp.. Appl. Environ. Microbiol..

[12] Tsavkelova E.A., Volynchikova E.A., Potekhina N.V., Lavrov K.V., Avtukh A.N. (2024). Auxin production and plant growth promotion by *Microbacterium albopurpureum* sp. nov. from the rhizoplane of leafless *Chiloschista parishii* orchid. Front. Plant Sci..

[13] Aniszewski E., Peixoto R.S., Mota F.F., Leite S.G.F., Rosado A.S. (2010). Bioemulsifier production by *Microbacterium* sp. strains isolated from mangrove and their application to remove cadmium and zinc from hazardous industrial residue. Braz. J. Microbiol..

[14] Tripathi R., Singh J., Bharti R.K., Thakur I.S. (2014). Isolation, purification and characterization of lipase from *Microbacterium* sp. and its application in biodiesel production. Energy Procedia.

[15] Qing Y., Tian J., Ma Z., Tang M., Long X. (2025). Taxonomic identification, genomic analysis, and optimized chromium(VI) bioreduction by *Microbacterium triticisoli* sp. nov. M28^T^. PeerJ.

[16] Hackmann T.J. (2025). Setting new boundaries of 16S rRNA gene identity for prokaryotic taxonomy. Int. J. Syst. Evol. Microbiol..

[17] Kim M., Oh H.-S., Park S.-C., Chun J. (2014). Towards a taxonomic coherence between average nucleotide identity and 16S rRNA gene sequence similarity for species demarcation of prokaryotes. Int. J. Syst. Evol. Microbiol..

[18] Riesco R., Trujillo M.E. (2024). Update on the proposed minimal standards for the use of genome data for the taxonomy of prokaryotes. Int. J. Syst. Evol. Microbiol..

[19] Richter M., Rosselló-Móra R. (2009). Shifting the genomic gold standard for the prokaryotic species definition. Proc. Natl. Acad. Sci. USA.

[20] Meier-Kolthoff J.P., Auch A.F., Klenk H.-P., Göker M. (2013). Genome sequence-based species delimitation with confidence intervals and improved distance functions. BMC Bioinform..

[21] Dashti A.A., Jadaon M.M., Abdulsamad A.M., Dashti H.M. (2009). Heat treatment of bacteria: A simple method of DNA extraction for molecular techniques. Kuwait Med. J..

[22] Frank J.A., Reich C.I., Sharma S., Weisbaum J.S., Wilson B.A., Olsen G.J. (2008). Critical evaluation of two primers commonly used for amplification of bacterial 16S rRNA genes. Appl. Environ. Microbiol..

[23] Chalita M., Kim Y.O., Park S., Oh H.S., Cho J.H., Moon J., Baek N., Moon C., Lee K., Yang J. (2024). EzBioCloud: A genome-driven database and platform for microbiome identification and discovery. Int. J. Syst. Evol. Microbiol..

[24] Kumar S., Stecher G., Suleski M., Sanderford M., Sharma S., Tamura K. (2024). MEGA12: Molecular Evolutionary Genetic Analysis Version 12 for adaptive and green computing. Mol. Biol. Evol..

[25] Saitou N., Nei M. (1987). The neighbor-joining method: A new method for reconstructing phylogenetic trees. Mol. Biol. Evol..

[26] Felsenstein J. (1981). Evolutionary trees from DNA sequences: A maximum likelihood approach. J. Mol. Evol..

[27] Fitch W.M. (1971). Toward defining the course of evolution: Minimum change for a specific tree topology. Syst. Zool..

[28] Kimura M. (1980). A simple method for estimating evolutionary rates of base substitutions through comparative studies of nucleotide sequences. J. Mol. Evol..

[29] Felsenstein J. (1985). Confidence limits on phylogenies: An approach using the bootstrap. Evolution.

[30] Chen S. (2025). fastp 1.0: An ultra-fast all-round tool for FASTQ data quality control and preprocessing. iMeta.

[31] Prjibelski A., Antipov D., Meleshko D., Lapidus A., Korobeynikov A. (2020). Using SPAdes de novo assembler. Curr. Protoc. Bioinform..

[32] Shen W., Sipos B., Zhao L. (2024). SeqKit2: A Swiss army knife for sequence and alignment processing. iMeta.

[33] Gurevich A., Saveliev V., Vyahhi N., Tesler G. (2013). QUAST: Quality assessment tool for genome assemblies. Bioinformatics.

[34] Parks D.H., Imelfort M., Skennerton C.T., Hugenholtz P., Tyson G.W. (2015). CheckM: Assessing the quality of microbial genomes recovered from isolates, single cells, and metagenomes. Genome Res..

[35] Seemann T. (2014). Prokka: Rapid prokaryotic genome annotation. Bioinformatics.

[36] Emms D.M., Kelly S. (2015). OrthoFinder: Solving fundamental biases in whole genome comparisons dramatically improves orthogroup inference accuracy. Genome Biol..

[37] Sievers F., Higgins D.G. (2018). Clustal Omega for making accurate alignments of many protein sequences. Protein Sci..

[38] Capella-Gutiérrez S., Silla-Martínez J.M., Gabaldón T. (2009). trimAl: A tool for automated alignment trimming in large-scale phylogenetic analyses. Bioinformatics.

[39] Nguyen L.-T., Schmidt H.A., von Haeseler A., Minh B.Q. (2015). IQ-TREE: A fast and effective stochastic algorithm for estimating maximum-likelihood phylogenies. Mol. Biol. Evol..

[40] Yoon S.-H., Ha S.-M., Lim J., Kwon S., Chun J. (2017). A large-scale evaluation of algorithms to calculate average nucleotide identity. Anton. Leeuw..

[41] Jain C., Rodriguez-R L.M., Phillippy A.M., Konstantinidis K.T., Aluru S. (2018). High throughput ANI analysis of 90K prokaryotic genomes reveals clear species boundaries. Nat. Commun..

[42] Meier-Kolthoff J.P., Carbasse J.S., Peinado-Olarte R.L., Göker M. (2022). TYGS and LPSN: A database tandem for fast and reliable genome-based classification and nomenclature of prokaryotes. Nucleic Acids Res..

[43] Blin K., Shaw S., Augustijn H.E., Reitz Z.L., Biermann F., Alanjary M., Fetter A., Terlouw B.R., Metcalf W.W., Helfrich E.J.N. (2023). antiSMASH 7.0: New and improved predictions for detection, regulation, chemical structures and visualisation. Nucleic Acids Res..

[44] Wishart D.S., Han S., Saha S., Oler E., Peters H., Grant J.R., Stothard P., Gautam V. (2023). PHASTEST: Faster than PHASTER, better than PHAST. Nucleic Acids Res..

[45] Mungan M.D., Blin K., Ziemert N. (2022). ARTS-DB: A database for antibiotic resistant targets. Nucleic Acids Res..

[46] Schleifer K.H., Kandler O. (1972). Peptidoglycan types of bacterial cell walls and their taxonomic implications. Bacteriol. Rev..

[47] Tang S.K., Wang Y., Chen Y., Lou K., Cao L.L., Xu L.H., Li W.J. (2009). *Zhihengliuella alba* sp. nov., and emended description of the genus *Zhihengliuella*. Int. J. Syst. Evol. Microbiol..

[48] Sasser M. (1990). Identification of bacteria by gas chromatography of cellular fatty acids. MIDI Tech. Note.

[49] Tamaoka J. (1986). Analysis of bacterial menaquinone mixtures by reverse-phase high-performance liquid chromatography. Methods Enzymol..

[50] Minnikin D.E., Collins M.D., Goodfellow M. (1979). Fatty acid and polar lipid composition in the classification of *Cellulomonas*, *Oerskovia* and related taxa. J. Appl. Bacteriol..

[51] Liu G.Y., Essex A., Buchanan J.T., Datta V., Hoffman H.M., Bastian J.F., Fierer J., Nizet V. (2005). *Staphylococcus aureus* golden pigment impairs neutrophil killing and promotes virulence through its antioxidant activity. J. Exp. Med..

[52] Thomashow L.S., Weller D.M. (1988). Role of a phenazine antibiotic from *Pseudomonas fluorescens* in biological control of *Gaeumannomyces graminis* var. *tritici*. J. Bacteriol..

[53] Shima S., Matsuoka H., Iwamoto T., Sakai H. (1984). Antimicrobial action of ε-poly-L-lysine. J. Antibiot..

[54] Griffiths G., Sigel S., Payne S., Neilands J.B. (1984). Vibriobactin, a siderophore from *Vibrio cholerae*. J. Biol. Chem..

[55] Canchaya C., Proux C., Fournous G., Bruttin A., Brüssow H. (2003). Prophage genomics. Microbiol. Mol. Biol. Rev..

[56] Feiner R., Argov T., Rabinovich L., Sigal N., Borovok I., Herskovits A.A. (2015). A new perspective on lysogeny: Prophages as active regulatory switches of bacteria. Nat. Rev. Microbiol..

[57] Collins J.A., Osheroff N. (2024). Gyrase and topoisomerase IV: Recycling old targets for new antibacterials to combat fluoroquinolone resistance. ACS Infect. Dis..

[58] Hong H.-J., Hutchings M.I., Buttner M.J., Utsumi R. (2008). Vancomycin resistance VanS/VanR two-component systems. Bacterial Signal Transduction: Networks and Drug Targets.

[59] Vaz-Moreira I., Lopes A.R., Falsen E., Schumann P., Nunes O.C., Manaia C.M. (2008). *Microbacterium luticocti* sp. nov., isolated from sewage sludge compost. Int. J. Syst. Evol. Microbiol..

[60] Lee H., Chaudhary D.K., Lee K.-E., Cha I.-T., Chi W.-J., Kim D.-U. (2024). *Microbacterium humicola* sp. nov., *Microbacterium terrisoli* sp. nov., *Paenibacillus pedocola* sp. nov., *Paenibacillus silviterrae* sp. nov., *Flavobacterium terrisoli* sp. nov., and *Aquabacterium humicola* sp. nov., isolated from soil. Int. J. Syst. Evol. Microbiol..

[61] Saticioglu I.B., Ajmi N., Coskuner-Weber O., Alpsoy S., Ay H., Aydin F., Abay S., Karakaya E., Kayman T., Dalyan C. (2025). Three new *Microbacterium* species isolated from the Marmara Sea mucilage event: *Microbacterium istanbulense* sp. nov., *Microbacterium bandirmense* sp. nov., *Microbacterium marmarense* sp. nov.. Syst. Appl. Microbiol..

