# Supplementary material for: Microbacterium altimontanum sp. nov., Microbacterium longlingense sp. nov., and Microbacterium cucumeris sp. nov.: Three New Species of Microbacterium Isolated from Soil in Yunnan Province, China
Source: Microorganisms. 2026 Jul 28;14(8):1644. doi: 10.3390/microorganisms14081644 (PMC13515409; doi:10.3390/microorganisms14081644)
Supplement: Supplementary file 1 [file microorganisms-14-01644-s001.zip › microorganisms-4448024-supplementary.pdf]

Microbacterium altimontanum sp. nov., _Microbacterium longlingense_ sp. nov., _Microbacterium cucumeris_ sp. nov., three new species of _Microbacterium_ isolated from soil in Yunnan Province, China.

Wenqi Lai [1,2,3], Xiankun Zhang[1,2,3], Zhongwen Duan[1,2,3], Mingji Yang[1,2,3], Yongxia Wang[4*], Zefen Yu[1,2*]

1 State Key Laboratory for Conservation and Utilization of Bio-Resources in Yunnan, Yunnan University, Kunming 650504, China

2 Yunnan Key Laboratory of Basic Research and Innovative Application for Green Biological Production, Yunnan University, Kunming 650504, China

3 School of Life Sciences, Yunnan University, Kunming 650504, China

4 Yunnan Institute of Microbiology, School of Life Sciences, Yunnan University, Kunming, 650504, China

Corresponding authors: Yongxia Wang (wangyx@ynu.edu.cn); Zefen Yu (zfyu@ynu.edu.cn)

This PDF file includes: 4 Figures and 6 Tables

Figure S1. Maximum-likelihood phylogenetic tree based on 16S rRNA gene sequences showing the relationships of strains AW10-10^T, M8-1^T and HGHZ1-2^T with members of the genus *Microbacterium*. Bootstrap values based on 1,000 replicates are shown at branch nodes when greater than 60%. *Micrococcus luteus* NCTC 2665^T was used as the outgroup. The scale bar represents 0.01 substitutions per nucleotide position.

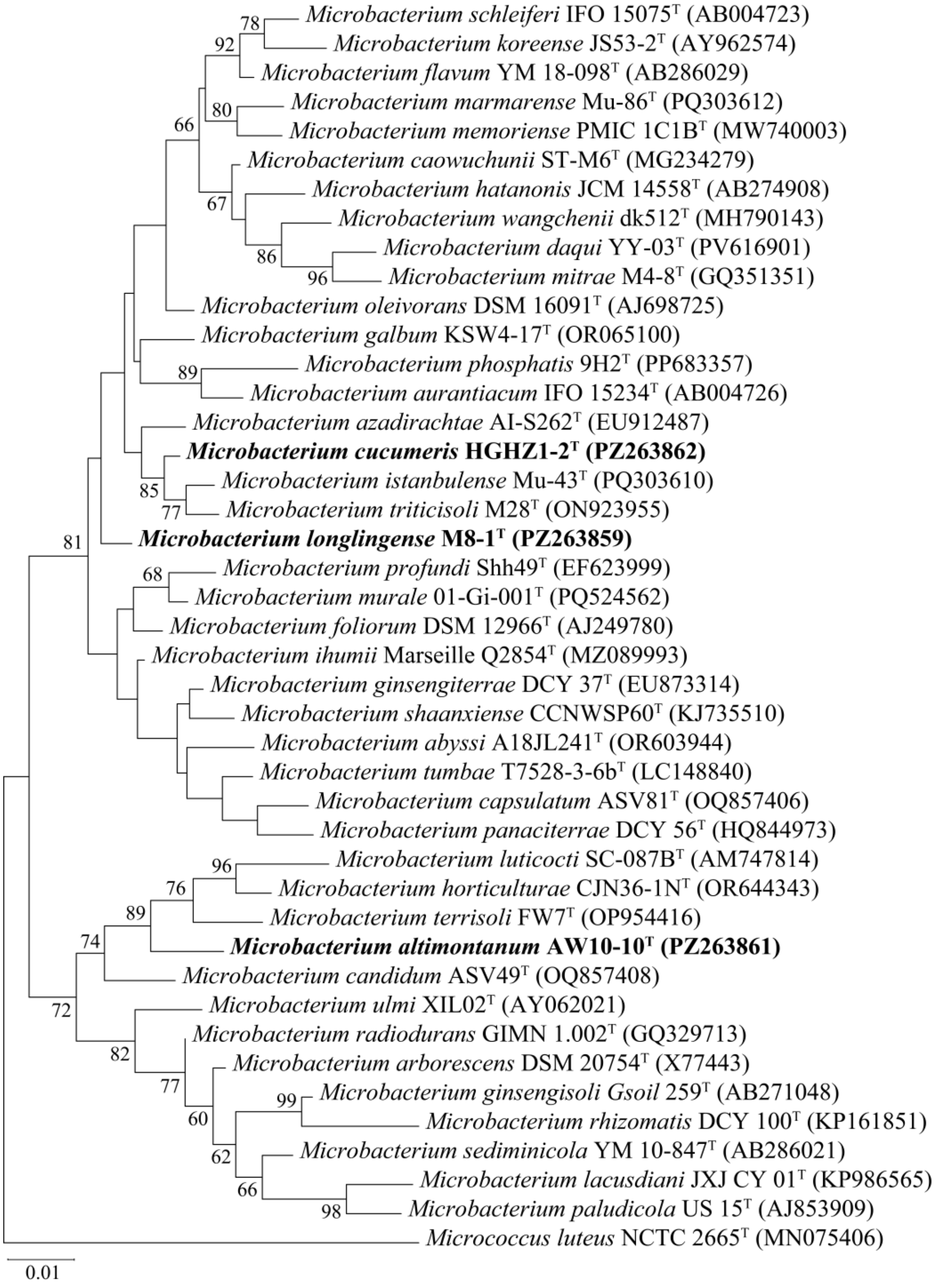

Figure S2. Maximum-parsimony phylogenetic tree based on 16S rRNA gene sequences showing the relationships of strains AW10-10^T, M8-1^T and HGHZ1-2^T with members of the genus *Microbacterium*. Bootstrap values based on 1,000 replicates are shown at branch nodes when greater than 60%. *Micrococcus luteus* NCTC 2665^T was used as the outgroup. The scale bar represents 20 nucleotide substitutions.

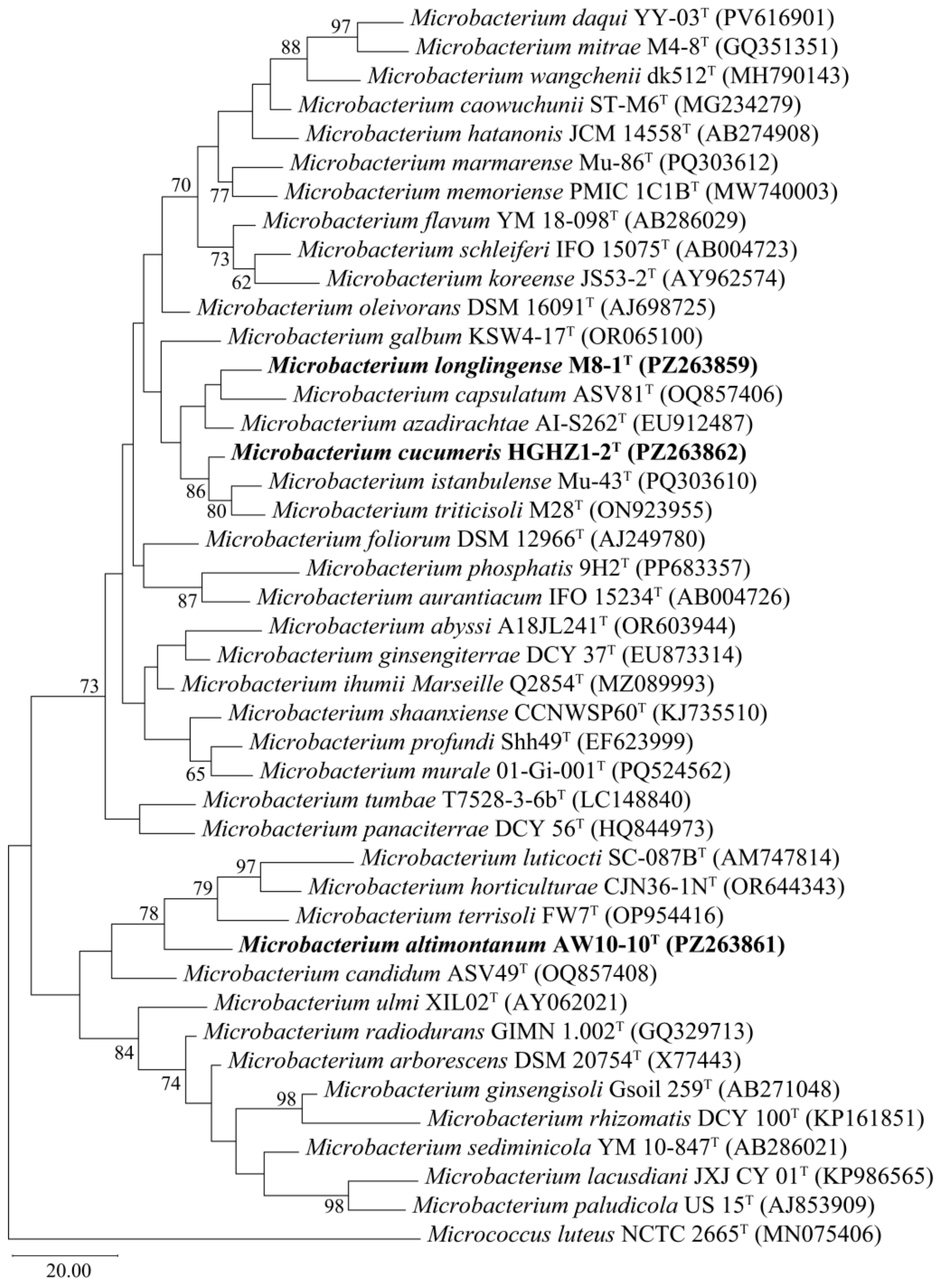

Figure S3. Transmission electron micrographs of strains AW10-10^T, M8-1^T and HGHZ1-2^T. Scale bars: 500 nm for AW10-10^T and M8-1^T, and 200 nm for HGHZ1-2^T.

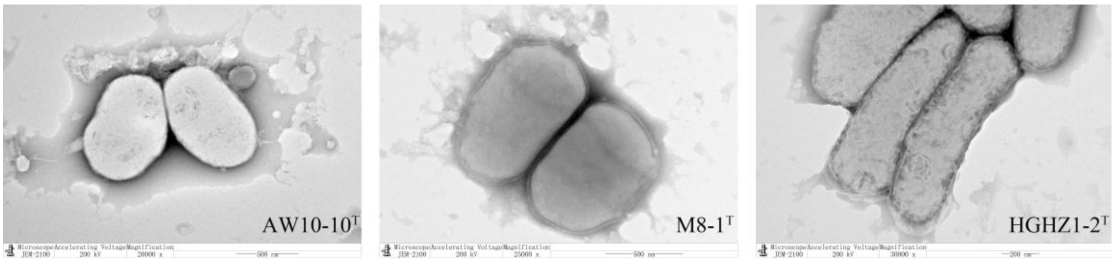

Figure S4. Two-dimensional thin-layer chromatograms of the polar lipids of strains AW10-10^T, M8-1^T, and HGHZ1-2^T.

(**a**) Total lipids; (**b**) phospholipids; (**c**) aminolipids; (**d**). glycolipids.

DPG, Diphosphatidylglycerol; PG, phosphatidylglycerol; GL, unidentified glycolipid.

AW10-10^T

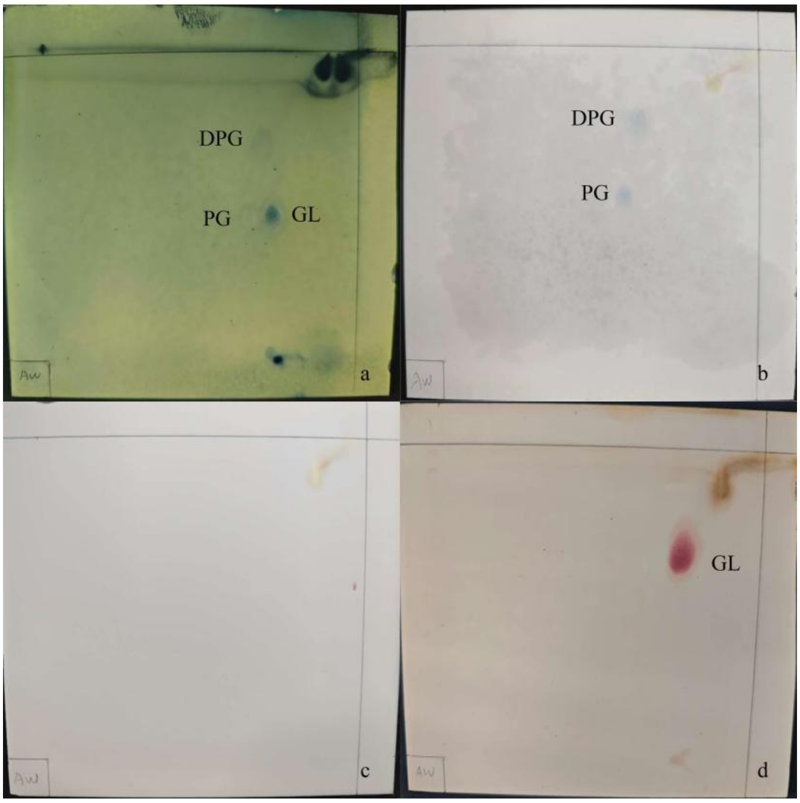

M8-1^{T}

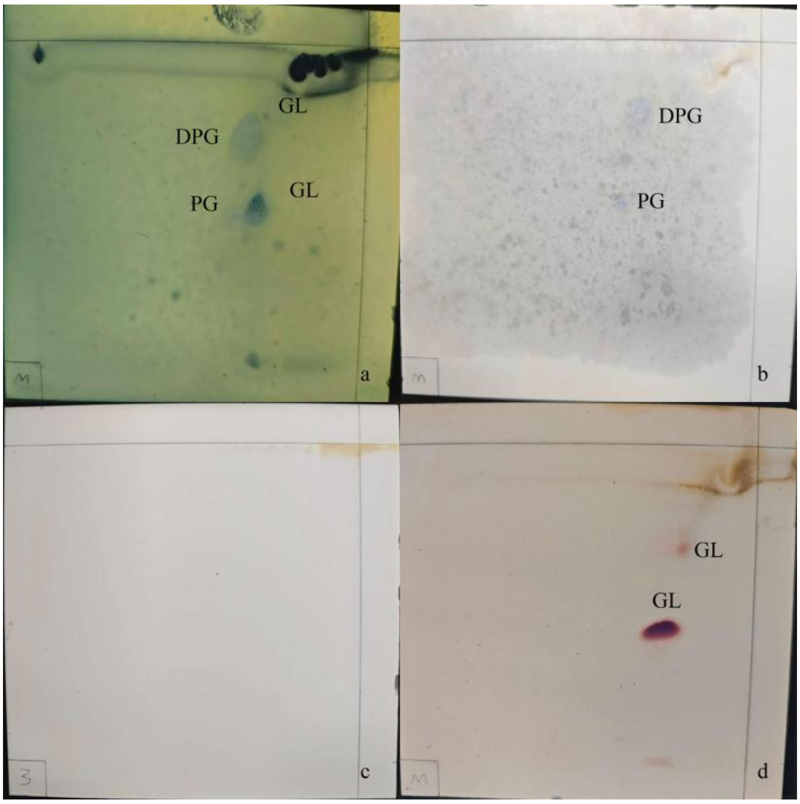

HGHZ1-2^{T}

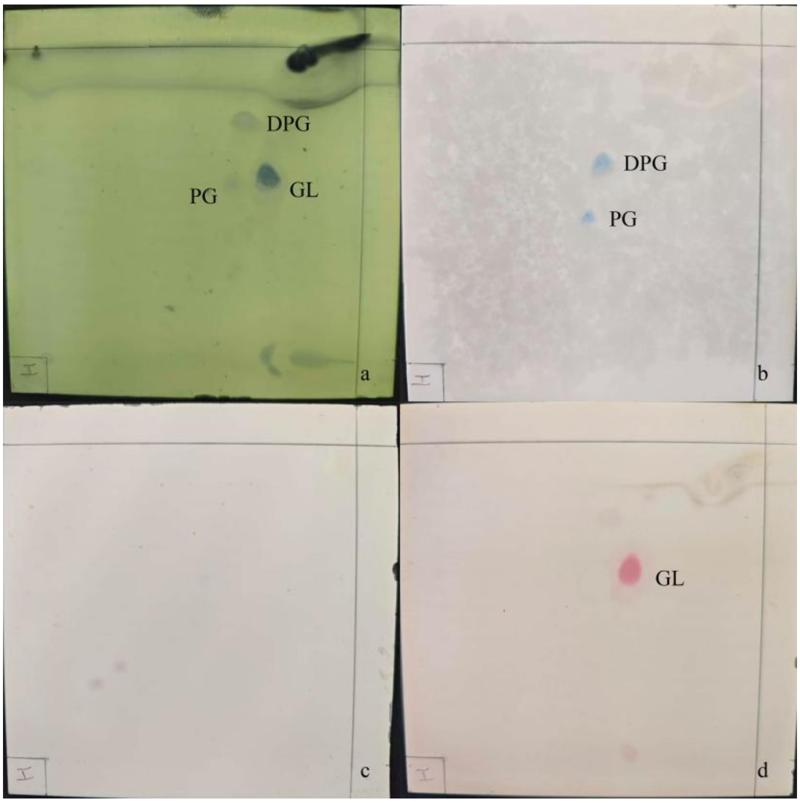

Table S1. 16S rRNA gene sequence similarities, average nucleotide identity (ANI) and digital DNA–DNA hybridization (dDDH) values among strains AW10-10^T, M8-1^T and HGHZ1-2^T and their five closest type strains.

Query Strain	Subject strain	16S rRNA gene sequence similarity (%)	OrthoANI (%)	FastANI	dDDH (%)
AW10-10^T	*M. mangrovi* MUSC 115^T	97.3	76.7	80.3	20.9
	M. ulmi CECT 5976^T	97.3	77.5	80.3	21.1
	M. immunditiarum DSM 24662^T	97.2	77.0	80.6	20.8
	M. protaetiae DFW100M-13^T	97.2	79.4	81.4	22.3
	M. horticulturae KACC 23027^T	97.1	79.6	81.9	22.5
	M. longlingense M8-1^T	96.2	75.0	79.1	20.8
	M. cucumeris HGHZ1-2^T	96.3	75.7	79.6	20.1
M8-1^T	*M. azadirachtae* CNUC13^T	98.6	76.1	80.2	21.2
	M. natoriense JCM 12611^T	98.3	76.2	80.0	21.3
	M. commune Re1^T	98.3	76.0	80.3	20.7
	M. resistens MZT7^T	98.3	76.3	79.6	21.1
	M. tenebrionis YMB-B2^T	98.3	76.6	80.1	21.1
	M. cucumeris HGHZ1-2^T	97.9	76.4	79.8	21.5
HGHZ1-2^T	*M. pullorum* Sa4CUA7^T	98.6	75.7	79.9	19.7
	M. resistens MZT7^T	98.5	77.5	80.9	21.2
	M. natoriense JCM 12611^T	98.5	78.6	81.6	22.2
	M. azadirachtae CNUC13^T	98.3	77.2	80.9	21.6
	M. keratanolyticum VKM Ac-1958^T	98.3	76.8	80.2	20.5

Table S2. Secondary metabolite biosynthetic gene clusters identified in strains AW10-10^T, M8-1^T, and HGHZ1-2^T using the antiSMASH.

Strain	Region	Type	Start(bp)	End(bp)	Most similar gene cluster	Similarity Confidence
**AW10-10^T**	Region2.1	phenazine	230,059	250,571	-	-
	Region4.1	terpene	223,882	244,790	carotenoid	Low
	Region6.1	T3PKS	153,280	194,464	5-acetyl-5,10-dihydrophenazine-1-carboxylic acid/5-(2-hydroxyacetyl)-5,10-dihydrophenazine-1-carboxylic acid/endophenazine A1/endophenazine F/endophenazine G	Low
	Region6.2	terpene-precursor	199,829	220,890	-	-
	Region6.3	terpene	290,504	315,699	carotenoid	Medium
	Region7.1	T3PKS	191,407	232,516	-	-
	Region9.1	terpene-precursor, betalactone	10,821	47,054	-	-
	Region9.2	RiPP-like	67,656	78,513	-	-
**M8-1^T**	Region2.1	Betalactone, terpene-precursor	116621	159635	-	-
	Region6.1	RiPP-like	35972	69901	ε-Poly-L-lysine	High
	Region6.2	T3PKS	241,362	282,504	5-acetyl-5,10-dihydrophenazine-1-carboxylic acid/5-(2-hydroxyacetyl)-5,10-dihydrophenazine-1-carboxylic acid/endophenazine A1/endophenazine F/endophenazine G	Low
**HGHZ1-2^T**	Region1.1	T3PKS, betalactone, terpene-precursor	35,822	89,040	vibriobactin	Low
	Region1.2	terpene	1,151,761	1,176,025	carotenoid	Low
	Region6.1	terpene-precursor	75,986	97,047	-	-

Table S3. A prophage region was identified in the genome of strain AW10-10^T using PHASTEST.

Strain	Contig	Region	Length	Completeness (score)	Region Position	Total Proteins	Most Common Phage	GC%
**AW10-10^T**	4	1	42.3Kb	Intact (150)	270575–312959	63	PHAGE_Microb_Min1_NC_009603(9)	67.7

Table S4. Antimicrobial resistance models were detected in the genomes of strains AW10-10^T, M8-1^T, and HGHZ1-2^T using the ARTS server.

Antimicrobial Resistance Model No	Model Description	AW10-10^T	M8-1^T	HGHZ1-2^T
RF0007	ABC_efflux	4	3	2
RF0033	APH3	2	1	1
RF0123	SubclassB1	1	0	0
PF00044.19	Gp_dh_N	2	2	1
PF00185.19	OTCace	2	2	2
PF00204.20	DNA_gyraseB	2	2	2
PF00364.17	Biotin_lipoyl	7	4	7
PF00521.15	DNA_topoisoIV	2	2	3
PF01039.17	Carboxyl_trans	2	1	2
PF13599.1	Pentapeptide_4	1	0	0
TIGR02013	TIGR02013	2	1	2
RF0002	AAC3	0	0	1
RF0051	Chlor_Efflux_Pump	0	1	1
RF0067	Erm23S_rRNA_methyltrans	0	1	1
RF0155	vanS	0	0	1
RF0053	ClassA	0	1	0
TIGR00663	TIGR00663	1	0	1

Table S5. Phenotypic characteristics of strains AW10-10^T, M8-1^T, and HGHZ1-2^T determined using Biolog GENIII, API 20NE, and API ZYM systems.

+; Positive, –; negative, W; Weak Positive

**Characteristics**		AW10-10^T	M8-1^T	HGHZ1-2^T
**Biolog GENIII**				
A2	Dextrin	+	-	+
A3	D-Maltose	+	+	+
A4	D-Trehalose	+	+	+
A5	D-Cellobiose	+	+	+
A6	Gentiobiose	+	+	+
A7	Sucrose	+	+	+
A8	D-Turanose	+	+	+
A9	Stachyose	W	W	+
B1	D-Raffinose	-	W	+
B2	α-D-Lactose	W	W	+
B3	D-Melibiose	W	W	+
B4	β-Methyl-D-Glucoside	W	+	+
B5	D-Salicin	+	+	+
B6	N-Acetyl-D-Glucosamine	W	W	+
B7	N-Acetyl-β-D-Mannosamine	+	W	+
B8	N-Acetyl-D-Galactosamine	+	W	+
B9	N-Acetyl-Neuraminic Acid	-	-	W
C1	α-D-Glucose	+	+	+
C2	D-Mannose	+	+	+
C3	D-Fructose	+	+	++
C4	D-Galactose	+	-	+
C5	3-Methyl Glucose	W	W	+
C6	D-Fucose	W	W	+
C7	L-Fucose	+	W	+
C8	L-Rhamnose	+	W	+
C9	Inosine	+	+	+
D1	D-Sorbitol	W	-	-
D2	D-Mannitol	+	-	+
D3	D-Arabitol	+	W	+
D4	myo-Inositol	-	W	W
D5	Glycerol	-	+	+
D6	D-Glucose-6-PO4	W	W	+
D7	D-Fructose-6-PO4	W	W	+
D8	D-Aspartic Acid	W	-	W
D9	D-Serine	W	W	-
E1	Gelatin	+	W	+
E2	Glycyl-L-Proline	+	W	W
E3	L-Alanine	W	+	+
E4	L-Arginine	W	W	W

E5	L-Aspartic Acid	+	-	W
E6	L-Glutamic Acid	+	-	+
E7	L-Histidine	W	W	+
E8	L-Pyroglutamic Acid	W	W	W
E9	L-Serine	+	+	+
F1	Pectin	+	+	+
F2	D-Galacturonic Acid	W	W	+
F3	L-Galactonic Acid Lactone	-	W	+
F4	D-Gluconic Acid	+	+	+
F5	D-Glucuronic Acid	W	W	+
F6	Glucuronamide	W	-	W
F7	Mucic Acid	W	-	W
F8	Quinic acid	W	W	W
F9	D-Saccharic Acid	-	-	-
G1	p-Hydroxy-Phenylacetic Acid	+	+	+
G2	Methyl Pyruvate	+	+	W
G3	D-Lactic Acid Methyl Ester	W	W	W
G4	L-Lactic Acid	+	+	+
G5	Citric Acid	-	+	-
G6	α-Keto-Glutaric Acid	W	-	W
G7	D-Malic Acid	W	-	-
G8	L-Malic Acid	W	W	W
G9	Bromo-Succinic Acid	-	-	-
H1	Tween 40	+	+	+
H2	γ-Amino-Butyric Acid	W	W	W
H3	α-Hydroxy Butyric Acid	+	W	W
H4	ß-Hydroxy-D,L-Butyric Acid	W	-	W
H5	α-Keto-Butyric Acid	W	+	+
H6	Acetoacetic Acid	W	+	+
H7	Propionic acid	+	+	W
H8	Acetic Acid	W	+	+
H9	Formic Acid	W	-	W
**Chemical sensitivity assays**				
A11	pH 6	+	+	+
A12	pH 5	+	-	-
B10	1% NaCl	+	+	+
B11	4% NaCl	+	+	+
B12	8% NaCl	-	+	+
C10	1% Sodium Lactate	+	+	+
C11	Fusidic Acid	-	W	-
C12	D-serine	-	+	-
D10	Troleandomycin	-	W	W
D11	Rifamycin SV	-	W	+
D12	Minocycline	-	W	-
E10	Lincomycin	-	-	-

E11	Guanidine HCl	W	-	W
E12	Niaproof 4	-	-	-
F10	Vancomycin	-	W	-
F11	Tetrazolium Violet	W	W	-
F12	Tetrazolium Blue	-	-	W
G10	Nalidixic Acid	+	+	-
G11	Lithium Chloride	+	+	+
G12	Potassium Tellurite	+	+	+
H10	Aztreonam	+	+	+
H11	Sodium Butyrate	+	+	+
H12	Sodium Bromate	-	W	-
**API 20NE**				
**NO3**	Reduction of Nitrate to Nitrite	+	-	+
**TRP**	Indole Production	-	-	-
**GLU**	Fermentation (D-Glucose)	-	-	-
**ADH**	Arginine Dihydrolase	-	+	-
**URE**	Urease	-	-	-
**ESC**	Hydrolysis of Aesculin	+	+	+
**GEL**	Hydrolysis of Gelatin	-	-	+
**PNPG**	β-galactosidase (Para-NitroPhenyl ßDGalactopyranosidase)	+	+	+
**Assimilation of:**				
**GLU**	D-Glucose	-	+	+
**ARA**	L-Arabinose	-	-	-
**MNE**	D-Mannose	+	+	+
**MAN**	D-Mannitol	+	+	+
**NAG**	N-acetyl-D-Glucosamine	-	+	-
**MAL**	D-Maltose	-	+	+
**GNT**	Potassium Gluconate	-	+	-
**CAP**	Capric Acid	-	-	-
**ADI**	Adipic Acid	-	-	-
**MLT**	Malic Acid	-	+	-
**CIT**	Citrate	-	+	-
**PAC**	Phenylacetic Acid	-	-	-
**API ZYM**				
2	Alkaline phosphatase	-	-	-
3	Esterase (C4)	+	+	+
4	Esterase lipase (C8)	+	+	+
5	Lipase (C14)	-	-	-
6	Leucine arylamidase	+	+	+
7	Valine arylamidase	+	+	+
8	Cystine arylamidase	+	+	+
9	Trypsin	+	+	+
10	Chymotrypsin	-	-	-
11	Acid phosphatase	+	+	+

12	Naphthol-AS-BI-phosphohydrolase	+	+	+
13	α-galactosidase	-	-	+
14	β-galactosidase	-	-	+
15	β-glucuronidase	-	-	-
16	α-glucosidase	+	+	+
17	β-glucosidase	+	+	+
18	N-acetyl-β-glucosaminidase	+	+	+
19	α-mannosidase	+	+	-
20	α-fucosidase	-	-	+

Table S6. Cellular fatty acid composition (%) of strains AW10-10^T, M8-1^T, and HGHZ1-2^T and their related type strains of the genus *Microbacterium*.

Strains: Strains: 1, AW10-10^T; 2, *M. luticocti* KACC 19377^T; 3, *M. horticulturae* CJN36-1N^T; 4, *M. terrisoli* FW7^T; 5, *M. candidum* ASV49^T; 6, M8-1^T; 7, HGHZ1-2^T; 8, *M. triticisoli* M28^T; 9, *M. azadirachtae* AI-S262^T; 10, *M. capsulatum* ASV81^T.

-, not detected; TR, trace amount (<1%). Values in bold indicate the three most abundant fatty acids in each strain.

**Fatty acid**	1	2	3	4	5	6	7	8	9	10
$C_{12:0}$	-	-	3.7	-	-	-	-	-	-	-
$C_{14:0}$	TR	-	1.3	TR	-	-	-	TR	-	-
$C_{16:0}$	3.0	-	8.2	1.0	-	3.7	3.4	1.2	-	2.1
$C_{18:0}$	-	-	4.4	-	-	-	-	-	-	-
iso-$C_{14:0}$	TR	-	4.1	2.5	1.4	TR	TR	2.3	TR	5.2
iso-$C_{15:0}$	3.5	**9.5**	1.2	1.3	5.4	2.1	1.8	11.7	10.1	4.8
iso-$C_{16:0}$	**16.7**	9.2	**17.8**	**36.8**	**21.3**	**16.3**	**21.1**	**28.4**	**18.0**	**44.0**
iso-$C_{17:0}$	2.8	3.0	-	1.9	5.1	-	2.5	5.0	2.7	1.4
iso-$C_{18:0}$	-	-	-	-	6.5	-	-	-	-	-
anteiso-$C_{14:0}$	-	-	2.2	-	-	-	-	-	-	-
anteiso-$C_{15:0}$	**19.0**	**32.9**	**24.3**	**41.2**	**38.8**	**54.6**	**22.9**	**35.6**	**45.9**	**24.5**
anteiso-$C_{17:0}$	**52.4**	**43.6**	**15.3**	**11.5**	**23.3**	**20.9**	**46.1**	**14.8**	**22.7**	**12.9**
$C_{15:1}$ ω8c	-	-	2.2	-	-	-	-	-	-	-
$C_{18:1}$ ω9c	1.3	-	1.9	-	-	1.6	1.0	-	-	-
iso-$C_{15:1}$	-	-	1.4	-	-	-	-	-	-	-
anteiso-$C_{15:1}$	-	-	TR	1.1	-	-	-	-	-	-
anteiso-$C_{17:1}$ ω9c	-	-	6.4	-	-	-	-	-	-	-
Summed features*										
4	-	-	-	-	-	-	-	-	-	1.6
8	TR	-	1.5	2.1	-	-	-	-	-	-

*Summed features are fatty acids that cannot be resolved reliably from another fatty acid using the chromatographic conditions chosen. The midi system groups these fatty acids together as one feature with a single percentage of the total. Summed feature 4 comprised iso-$C_{17:1}$ I and/or anteiso-$C_{17:1}$ B, whereas summed feature 8 comprised $C_{18:1}$ ω7c and/or $C_{18:1}$ ω6c.
